# Supplementary material for: Transcription factor competition facilitates self-sustained oscillations in single gene genetic circuits
Source: PLoS Comput Biol. 2023 Sep 29;19(9):e1011525. doi: 10.1371/journal.pcbi.1011525 (PMC10566692; doi:10.1371/journal.pcbi.1011525)
Supplement: Attachment — Submitted filename: ResponseReviewers.pdf [file pcbi.1011525.s003.pdf]

Utrecht, February 17 2023

Dear Editor,

We submit our manuscript “Transcription factor competition facilitates self-sustained oscillations in single gene genetic circuits" for publication in PLOS Computational Biology. This is a rigorously rewritten and significantly extended version of an earlier (and rejected) submission (PCOMPBIOL-D-21-02150). Our main point remains that in the simplest possible genetic feedback architecture, oscillations are possible in the absence of cooperative binding of transcription factor (TF) molecules to promoter sites, provided there are multiple, competing sites for TF binding. In fact we were pleased with and motivated by the very constructive comments of the reviewers. We quote the last remark of Reviewer 1: “*I hope these comments will help the authors to improve the presentation of their very important work.”* This and several other comments and questions, to be addressed in detail below, greatly encouraged us to put our work in a (much) broader perspective. After discussions with colleagues (in particular with dr. Aaron New, see Acknowledgement) and diving into the literature again, we found that our model is in fact similar to the classical “Goodwin oscillator” which is currently seen as a general feedback model in biology (see Ref. 68 in our manuscript). At the same time we introduced from more basic considerations the statistical mechanics that underlies our model, and put a lot more effort in explaining the definitions (e.g., of the fold change function). We apologize in hindsight for the way too technical and dense writing style in our previous version, which indeed led to understandable confusion of the reviewers. Finally, we compare our results to experiments we found in the literature. This, we believe, addresses the main point of Reviewer 2, that is, (quote) “..the applicability of this method to actual gene expression data is neither demonstrated or clearly evident”.

In the following we address point by point the comments and questions of the reviewers.

We hope you will consider our manuscript for publication in PLOS Computational Biology.

Yours sincerely,

Jasper Landman, Sjoerd Verduyn Lunel and Willem Kegel.

Reviewer 1: This paper presents a novel approach to the question of mechanistic requirements for sustained oscillations in genetic regulatory mechanisms. The authors show that oscillations are possible in the absence of cooperative binding of transcription factor (TF) molecules to promoter sites, provided there are multiple, competing sites for TF binding. The methodology by which the authors reach their conclusions seems to be sound, and their results are interesting, important and novel. The paper certainly deserves publication in PLoS CB.

Reply: We would like to thank the reviewer for this very positive evaluation of the importance of our results.

Reviewer 1: However, there are some serious weaknesses with the paper that need to be addressed in a revised version before the work is suitable for publication.

In my opinion, the paper is not clearly written and the authors do not provide enough information for interested readers to fully understand and adequately reproduce the main results. The paper is certainly 'not accessible to non-specialists' and it is a 'hard slog' even for a specialist such as myself. The main problem is that the authors never clearly define (in computational terms) the 'fold-change' function that appears in Eq. (8) and in the examples that follow. True, the fold-change function seems to be defined in Eq. (10)—for the case of multiple copies of a gene that encodes its own repressor (P)—in terms of the fugacity, λ_P, of the repressor protein. But the authors don't provide a prescription for calculating λ_P, so it is impossible for an interested reader to reproduce the results in Figure 2 or any of the other figures.

To try to understand how to use Eq. (8), I turned to the authors' 2017 PLoS One paper, where the 'weak promoter' limit is derived for the case of a single-copy gene for which P binds to multiple non-specific DNA-binding sites: $FC = \frac{1}{1+x_p\left(\frac{P}{N_{ns}}\right)}$, where P = copy number of the repressor, N_{ns} = number of non-specific sites, and x_P = constant (dependent on the binding-energy of the repressor to the DNA sites). Problem is: this is not the case in Figure 2 of the present paper, for which N_{ns} = 1, and N = number of gene copies >>1. The case of multiple copies of a regulated gene is not treated in the PLoS One paper (as far as I can see), so how am I to confirm the results in Figure 2 for N = 10 and 1000? Is there not some reasonably simple function for FC(P) for the case considered in Figure 2? FC appears to be a sharp sigmoidal function of P for N large; how does it compare with a Hill function, $H = \frac{1}{1+\left(\frac{P}{K}\right)^n}$, with two adjustable parameters n and K?

The case in Figure 3 (single gene + multiple, non-specific binding sites) is covered by the FC function derived in the 2017 paper. But now I am really confused, because $FC = \frac{1}{1+x_p\left(\frac{P}{N_{ns}}\right)} = \frac{1}{1+\left(\frac{P}{K}\right)}$ is a simple hyperbolically decreasing function of P for any value of N_{ns}, i.e., a 'Hill function' with exponent = 1. I don't think the model can oscillate in this case. The steady state is $2P^* = -K + \sqrt{K^2 + 4K}$ and $-\frac{dFC}{dP}(P^*) = K\left(\frac{P^*}{K}\right)^2 = 1 - \frac{K}{2}\left(\sqrt{1+\frac{4}{K}} - 1\right) < 1$, for all $K \geq 0$.

For this case, the slope of FC at the steady state lies between 0 and -1, so I don't see how this model can oscillate.

I am sure the problem is that I am not calculating FC(P) correctly, but the authors don't provide any help for an interested reader (or a reviewer) to reproduce the results in this paper or extend the method to other situations.

At the very least, the authors must provide matlab code to reproduce the results in their figures. Then an interested reader could figure out how this methodology is applied to practical problems. Without a template for applying their methodology to typical gene control circuits, the method is nearly impossible for anyone else to adopt.

Reply: We apologize for the much too technical writing style. Reviewer 1 went way beyond expectations in their efforts to reproduce our findings, but hit a serious flaw in the description of our method as we presented it. We greatly appreciate these efforts.

It is clear that we should have included far more detail to the readers and reviewers to allow modelers to reproduce our findings and to use the methods we present in other circumstances. The manuscript has undergone a rigorous rewriting. We have implemented the following changes addressing the comments of the reviewer:

Implemented changes to the manuscript:

- A full new section IIA has been added to the manuscript. This section presents a thermodynamic background and derivation of the grand canonical model used. In particular, it shows that the nonlinearities leading to locally increased slopes in the fold-change function come from sharp transitions in the transcription factor fugacity, arising from the titration effect.
- We also introduced a new illustrative figure that shows the statistical weights of the configurational states in both the canonical ensemble (traditionally used for equilibrium transcription factor models) as well as in the grand canonical ensemble.
- Furthermore, we have introduced a supplementary information section S2 that provides a step by step guide to calculate the transcription factor fugacity for all cases treated in this manuscript. Following the steps in this section leads to closed expressions for the fold-change as a function of transcription factor copy number.
- Finally, an annotated Mathematica notebook is provided as supplementary material, which can reproduce all the figures in the manuscript.

Reviewer 1: In Eq. (1), n should be referred to as the Hill exponent. It is not a coefficient, even though it is commonly/mistakenly called so.

Implemented changes to the manuscript:

- The parameter *n* is now called Hill exponent throughout the manuscript.

Reviewer 1: 'Hill isotherm' and 'Langmuir isotherm' are distracting jargon. The 'Langmuir isotherm' (case n = 1) is a simple rectangular hyperbola; why not call it such. The 'Hill isotherm' is just the Hill function; why give it two names?

Reply: We agree about the use of Hill function, and have changed the text accordingly. We have chosen to keep the term Langmuir isotherm, as this function has a distinct physical interpretation and historical importance.

Implemented changes to the manuscript:

- The name Hill Isotherm was changed to Hill function throughout the manuscript.

Reviewer 1: Figure 1a introduces two crucial ideas that are not defined in the text or legend: a 'fold-change' function and a 'grand canonical ensemble'. How is the reader to make sense of this figure? The 'fractional occupancy' θ is defined, and makes perfect sense. 'Why does the Hill function fit θ(P) but not FC(P)?' the reader is bound to wonder...but the authors don't provide any explanation that I could find. Figure 1c introduces another undefined term, 'fugacity'. As I recall from PChem class decades ago, the dimensionless fugacity coefficient is a 'fudge factor' to make 'real solutions' look like 'ideal solutions'. What does it have to do with TF binding to DNA in the presence of competitive binding sites? Can't the authors give the reader some help here?

Reply: The reviewer is correct – the figure appeared in the original manuscript well before the appropriate definitions were made. In the newly introduced section IIA, the grand canonical partition function is introduced, as is the fugacity. Furthermore, we found upon further consideration that the figure itself would be better placed in the supporting information. There a small additional paragraph was placed to explain the meaning of the figure.

Implemented changes to the manuscript:

- Figure 1 was moved to the supplementary information (S1), along with a small paragraph providing more information about the figure.
- The new section IIA introduces the grand canonical ensemble, the fugacity, the occupational fraction and the fold-change function.

Reviewer 1: 'We can use expressions for the fold-change of genes derived in the grand canonical ensemble...' Yes, the authors can use these expressions to present very intriguing results, but the authors don't help their readers to do likewise. Please show us (with matlab code) how to do so ourselves!

Implemented changes to the manuscript:

- We have provided an annotated Mathematica notebook that can reproduce all the figures.

Reviewer 1: In Eqs. (2, 3, 8, 9) the authors use matrix notation for ODEs when the rate-constant matrices are diagonal. In this case, the matrix notation is counter-productive. It's better to write the ODEs in component form, e.g.

$$\frac{\mathrm{d}P_i}{\mathrm{d}t} = \beta_i M_i - \gamma_i P_i, \frac{\mathrm{d}M_i}{\mathrm{d}t} = F_{i(P_{1(t-\tau)},\ldots,P_{N(t-\tau)})} - \delta_i M_i, i = 1, \ldots, N$$

where N = number of genes in the gene regulatory network and F_i = rate function for transcription of the ith gene as a function of the time-delayed concentrations of the regulatory proteins.

Reply: The reviewer points out that the notation could be made easier by writing the DDEs in component form. Here, we have decided to compromise somewhat on readability in favour of mathematical consistency and accuracy of notation, and keep the matrix notation of the system of DDEs. We have made this choice in particular since the vector **k** depends on the concentration of all proteins **P**, and this dependency causes the matrices to not be strictly diagonal anymore. We are aware of the legibility issues this causes, particularly because we make a detour through the component form to get from the original set of DDEs in eq. (11,12) to the normalized set of DDEs in eqs. (15).

Implemented changes to the manuscript:

- To improve legibility, we have revised our notation throughout the manuscript for better consistency and mathematical accuracy.

Reviewer 1: Above Eq. (3) the authors claim that the degradation rate constants for mRNAs and proteins are roughly the same. But in most organisms, mRNAs are much less stable than proteins. Otherwise, gene regulation wouldn't work very well...protein synthesis can't be shut off if mRNAs are stable.

Reply: The reviewer is correct that mRNAs tend to be less stable than proteins, and we apologise for this inaccuracy. As we show later in the manuscript, we do not need to assume these rate constants to be equal or even to be on the same order of magnitude for the models presented in this manuscript to work.

Implemented changes to the manuscript:

- In line 346, the paragraph about degradation rates of mRNA and protein has been amended to "The mRNA degradation rate constants are typically higher in magnitude compared to the protein rate constants Γ_P. However, often the dominant mechanism causing first order decay is dilution due to a global growth rate, in which case the mRNA and protein degradation rates are comparable [15]. In the latter case, active degradation mechanisms may still alter the individual degradation rate constants."

Reviewer 1: Finally, in Eq. (6) the authors define the fold-change function $FC_i\left(P_{1(t-\tau)}, \dots, P_N(t-\tau)\right) = \theta_i(P\_1(t-\tau), \dots, P\_N(t-\tau))/\theta_i^0$ where $\theta\ (P\ , \dots, P\ )$ is the fractional occupancy of RNAP at the promoter region of the ith gene in the regulated network, and θ^0 is the same for the unregulated network. (By the way, why is the time-delayed fraction called the 'instantaneous' fold-change?) The authors should introduce some appropriate notation for the fold-change function, instead of spelling out 'fold-change' whenever referring to it.

Implemented changes to the manuscript:

- We introduced the symbol Φ as the fold-change function, and $\mathbf{\Phi}$ as its vector form.
- On page 3, line 277, we added a paragraph to the new section IIA that introduces the fraction $\theta_1(\lambda_1, \lambda_2, \dots)\ /\ \theta_1(\lambda_1, 0, \dots)$ and discusses in what conditions this fraction can be assigned the physical interpretation fold-change.
- On page 5, line 390, rather than using the physical interpretation of the fold-change to identify Φ in the DDE of eq. (14), we directly use the fraction $\theta_1(\mathbf{P})/\ \theta_1(0)$ to make this identification, connecting it to its definition in section IIA.

Reviewer 1: Below Eq. (7) the authors refer to γ^i. The 'ce' is a typo, I presume. A few lines later, they normalize the copy numbers 'by their steady-state copy numbers'. I think they mean to say, 'by their unregulated steady-state copy numbers' (P^0 and M^0 in my simpler notation).

Reply: We thank the reviewer for pointing out these typos and inaccuracies in the text. We have amended them as advised.

Implemented changes to the manuscript:

- In line 389 the text was changed to "steady-state unregulated copy numbers of (…)"
- The notation of the rate constants was changed throughout the manuscript.

Reviewer 1: Last line of p 9: 'repressor quickly proceed to a stable steady-state value' would be more precise.

Implemented changes to the manuscript:

- In line 440, the text was amended to "In fig. 2 we see that for a low copy number of genes, the concentrations of mRNA and repressor quickly proceed to a stable steady state, (…)"

Reviewer 1: Page 14: ‘As a consequence, a gene without a cooperative architecture will be unable to sustain oscillatory behavior...’ Right; this is why I am confused by the authors’ example of a single gene with multiple competing sites (Figure 3). In the PLoS One paper, they show that the FC function has n = 1, in the case 1 << P << N_c. Why is the case $N_c = 30$ (Figure 5d) so much different?

Reply: We believe that the confusion of the reviewer stems from the claim in the 2017 PLOS One paper which states that for 1 << P << N_{ns} the fugacity of the repressor protein approaches P/N_{ns}. We realise that we have been somewhat imprecise with the text in the 2017 PLOS One paper. For this equivalence to hold, the repressor copy number needs to be significantly higher than the number of specific sites N and competitor sites N_c to hold. When the repressor copy number is comparable or lower than either, the titration effect will take place and the resulting fugacity is significantly lower.

The more significant the titration effect (due to either multiple gene copies N or competitor sites N_c), the sharper the transition between non-saturation and saturation of the binding sites. Only when there exists a region of the fold-change function that is steeper than the stability contour, there will be a regime where sustained oscillations are possible. This is why in Fig. 5d the case for $N_c = 30$ is different than for a lower number competitor sites. (in fact, already for $N_c = 21$, there is a small region where the slope of the fold-change function crosses the stability contour).

We hope that with the new section IIA, which provides much more insight into the meaning of the fugacity and the nature of the titration effect, this confusion is relieved.

Implemented changes to the manuscript:

- The new section IIA provides details on the nature of the fugacity and its interpretation.

Reviewer 1: I hope these comments will help the authors to improve the presentation of their very important work.

Reply: We would like to thank the reviewer with their efforts to understand our paper. It is clear in hindsight that we should have provided much more information. We believe that the significant rewrite has markedly improved the quality of this manuscript.

Reviewer 1: No: The authors must provide code (preferably matlab) to reproduce all the results in this paper. Otherwise, their results are useless to other modelers.

Implemented changes to the manuscript:
A new annotated Mathematica notebook is provided in the supplementary materials that reproduces all the figures in the manuscript.

Reviewer 2: The authors demonstrate how the mathematical modeling of mechanistic fold-change functions via delay-differential equations can model genetic feedback loops specifically with in regards to self-sustained oscillatory behavior. While the premiss is interesting, the presentation of the work is difficult to follow at best. Key terms such as grand canonical formalism are used repeatedly without first being defined, and a number of biological properties of the systems are stated as true without citations (and which are not globally accurate).

Reply: We apologise for the dense presentation of our work. In response, we have significantly rewritten our manuscript to be much clearer about the way the model works. In particular, we have introduced a new section IIA that repeats the thermodynamic basis of the grand canonical model we presented before in our 2017 PLOS One paper. In addition, supplementary materials have been provided that assist the reader in calculating the fugacity and the titration effect, as well as a Mathematica notebook with annotations that can reproduce all the figures in the manuscript.

The reviewer also mentions biological properties of the system that are states as true without citations. This likely refers to the claim that degradation rate constants for mRNA and protein are of comparable magnitude. We apologise for this inaccuracy and have amended the paragraph as detailed below.

We think that with the rewrite, our manuscript is significantly easier to follow.

Implemented changes to the manuscript:

- A full new section IIA has been added to the manuscript. This section presents a thermodynamic background and derivation of the grand canonical model used. In particular, it shows that the nonlinearities leading to locally increased slopes in the fold-change function come from sharp transitions in the transcription factor fugacity, arising from the titration effect.
- We also introduced a new illustrative figure that shows the statistical weights of the configurational states in both the canonical ensemble (traditionally used for equilibrium transcription factor models) as well as in the grand canonical ensemble.
- Furthermore, we have introduced a supplementary information section S2 that provides a step by step guide to calculate the transcription factor fugacity for all cases treated in this manuscript. Following the steps in this section leads to closed expressions for the fold-change as a function of transcription factor copy number.
- Finally, an annotated Mathematica notebook is provided as supplementary material, which can reproduce all the figures in the manuscript.
- In line 346, the paragraph about degradation rates of mRNA and protein has been amended to “The mRNA degradation rate constants are typically higher in magnitude compared to the protein rate constants Γ_P. However, often the dominant mechanism causing first order decay is dilution due to a global growth rate, in which case the mRNAand protein degradation rates are comparable [15]. In the latter case, active degradation mechanisms may still alter the individual degradation rate constants.”

Reviewer 2: The criteria by which it is possible to predict whether a given genetic feedback circuit will lead to self-sustained oscillations is of particular interest; however, the applicability of this method to actual gene expression data is neither demonstrated or clearly evident. In all this work raises interesting implications, but would be better suited to a more specialized journal in its current form.

Reply: We agree that direct comparison to experiments would significantly strengthen the claims we put forward in our manuscript. In the meantime we have been able to find a study that can at least be semi-quantitatively explained by our model. We have added a new section IIIE that compares experiments presented by Stricker et al. (Nature 2008) with our model. In the work of Stricker et al., an assay is presented with only a single direct feedback loop which oscillates when induced with IPTG. When a 3x excess of IPTG is added, the oscillations are once again suppressed. Our model reproduces this behaviour when IPTG is modelled as a competitive binding site for the repressor.

Implemented changes to the manuscript:

- A new section IIIE was added to compare our model to experimental data presented in Stricker et al. (Nature 2008).
- Supplementary section S5 was introduced to detail how experimental parameters needed in the model were estimated from various sources.
