# Supplementary material for: Transcription factor competition facilitates self-sustained oscillations in single gene genetic circuits
Source: PLoS Comput Biol. 2023 Sep 29;19(9):e1011525. doi: 10.1371/journal.pcbi.1011525 (PMC10566692; doi:10.1371/journal.pcbi.1011525)
Supplement: Attachment — Submitted filename: for PLOS CB.pdf [file pcbi.1011525.s004.pdf]

Ultrasensitivity by protein sequestration

References

Buchler & Louis (2008) J Mol Biol 384:1106
Buchler & Cross (2009) Mol Sys Biol 5:272
Kim & Forger (2012) Mol Sys Biol 8:630

Theory

Transcr factor R binds to UTR of gene D to regulate production of mRNA M, which encodes protein P, which promotes production of R. In inhibitor I binds to R to pull R away from gene D.

R + D ↔ C_{RD}, K_D = effective dissoc const

R + I ↔ C_{RI}, K_I = effective dissoc const

Governing ODEs:

$$\frac{dM}{dt} = k_{sm}\frac{D}{D_T} - k_{dm}M$$

$$\frac{dP}{dt} = k_{sp}M - k_{dp}P$$

$$\frac{dR}{dt} = k_{sr}P - k_{dr}R - k_{bri}R \cdot I + k_{dri}C_{RI} - k_{brd}R \cdot D + k_{drd}C_{RD}$$

$$\frac{dC_{RI}}{dt} = k_{bri}R \cdot I - k_{dr}C_{RI} - k_{dri}C_{RI}$$

$$\frac{dC_{RD}}{dt} = k_{brd}R \cdot D - k_{dr}C_{RD} - k_{drd}C_{RD}$$

Let $R_T = R + C_{RI} + C_{RD}$. Then

$$\frac{dR_T}{dt} = k_{sr}P - k_{dr}R_T$$

Assume fast, reversible complex formation:

$$K_I C_{RI} = R \cdot I = (R_T - C_{RI} - C_{RD})(I_T - C_{RI});\ K_I = \frac{k_{dri} + k_{dr}}{k_{bri}}$$

$$K_D C_{RD} = R \cdot D = (R_T - C_{RI} - C_{RD})(D_T - C_{RD});\ K_D = \frac{k_{drd} + k_{dr}}{k_{brd}}$$

We expect that $D_T \ll I_T$ and $C_{RD} \ll C_{RI}$. In that case,

$$C_{RI} = \frac{1}{2}\left(R_T + I_T + K_I + \sqrt{(R_T + I_T + K_I)^2 - 4R_T \cdot I_T}\right)$$

and

$$K_D C_{RD} = (R_T - C_{RI})(D_T - C_{RD});\ \text{i.e.,}\ \frac{C_{RD}}{D_T} = \frac{R_T - C_{RI}}{K_D + R_T - C_{RI}}$$

So

$$\frac{D}{D_T} = \frac{K_D}{K_D + R_T - C_{RI}} = \frac{2K_D}{2K_D + R_T - I_T - K_I + \sqrt{(R_T + I_T + K_I)^2 - 4R_T \cdot I_T}}$$

Simulate the model using XPP with this input file:

```
# Negative Feedback Oscillations
# Nonlinearity provided by protein sequestration

dM/dt = ksm*D/DT - kdm*M
dP/dt = ksp*M - kdp*P
dRT/dt = ksr*P - kdr*RT

D = 2*KD*DT/(2*KD*DT+RT-IT-KI+sqrt((RT+IT+KI)^2-4*RT*IT))

par ksm=3000, ksp=1, ksr=1, kdm=1, kdp=1, kdr=1
par IT=100, DT=1, KD=0.05, KI=0.1
init M=35, P=70, RT=100
@ xlo=0, ylo=0, xhi=20, yhi=200, bounds=1000
done
```

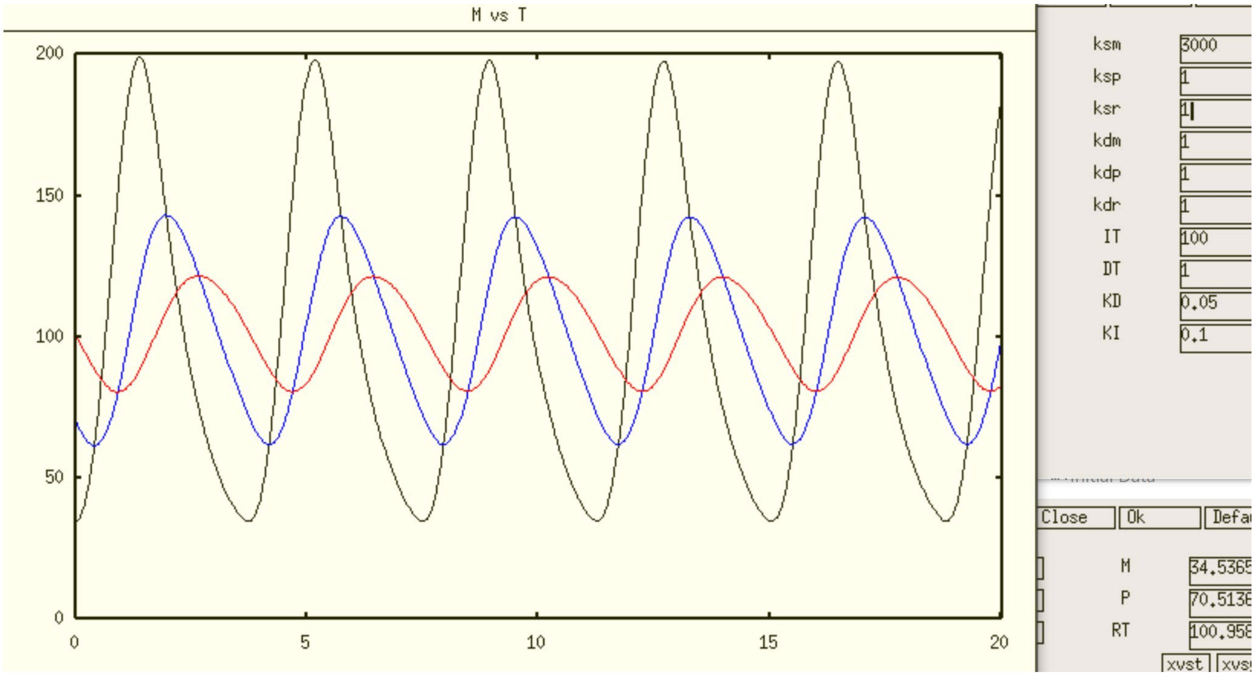

Black = M, blue = P, red = RT
