# Supplementary material for: Transcription factor competition facilitates self-sustained oscillations in single gene genetic circuits
Source: PLoS Comput Biol. 2023 Sep 29;19(9):e1011525. doi: 10.1371/journal.pcbi.1011525 (PMC10566692; doi:10.1371/journal.pcbi.1011525)
Supplement: Attachment — Submitted filename: Review of PCOMPBIOL 23-00261R1.pdf [file pcbi.1011525.s006.pdf]

Review of PCOMPBIOL-D-23-00261R1

The authors have adequately addressed all of the comments and suggestions of the Reviewers, so I am willing to recommend publication. However, I have a few comments for the authors and the editor, which may require some minor changes.

The authors make clear in their response to me that their complaint is with using a Hill function to describe the effect of TF sequestration, not with using standard biochemical reaction kinetic equations. I agree with them that Hill functions are not appropriate in this context, but I would argue that the standard kinetic approach is much simpler than the grand canonical approach introduced by the authors. There is no difficulty with applying the standard approach to more complex systems with multiple species involved in many binding reactions: standard numerical methods of solving differential algebraic equations (DAEs) handle such problems easily and accurately. I don't understand how linking the grand canonical approach to 'mechanistic adsorption problems' is an advantage for computational systems biologists. I don't think the authors provide sufficient motivation to readers to learn the methodology of grand canonical ensembles. If standard kinetic rate equations are OK, why make life difficult?

This is one case in which I think it may be advantageous to publish an addendum to the paper, making public some of the interchanges between the authors and reviewers (suitably edited).

Regarding the change proposed to the paragraph starting on line 88:

Although the notion exists that a competition-driven titration effect can also lead to sufficient nonlinearity to generate self-sustained oscillating reactions --- such circuits are coined `titration oscillators'[31-34] --- equilibrium modelling of transcription factor binding in these circumstances has so far resorted to a mechanistically incorrect mechanism: namely allosteric cooperative binding for which the Hill equation was derived.[35-40]. Kim & Tyson [41] level similar criticism at the use of quasi-Steady State Michaelis Menten kinetics which also reduces to a Hill equation."

I don't agree with the blue change. Although I haven't checked all references 35-40, I don't think the authors actually <u>derived</u> a Hill function from 'equilibrium modeling' of TF binding, but rather they <u>assumed</u> that a Hill function would adequately describe the situation. This assumption may be mechanistically incorrect, but other more recent papers [31-34, 41] apparently use singular perturbation methods to get mechanistically correct and accurate approximations for 'titration effects'. I think the authors should be more accurate in what they say about these papers [31-40], especially so when leveling criticisms.
