# Supplementary material for: Transcription factor competition facilitates self-sustained oscillations in single gene genetic circuits
Source: PLoS Comput Biol. 2023 Sep 29;19(9):e1011525. doi: 10.1371/journal.pcbi.1011525 (PMC10566692; doi:10.1371/journal.pcbi.1011525)
Supplement: Attachment — Submitted filename: ResponseReviewers_20230801.docx [file pcbi.1011525.s005.docx]

Utrecht, August 7, 2023

Dear professor Mendes,

We were pleased to receive the very constructive referee reports from the three referees on our manuscript PCOMPBIOL-D-23-00261. Below we’ve listed the referee responses, along with our response and corresponding changes in the manuscript. With these changes we hope that our manuscript is now acceptable for publication in PLOS Computational Biology.

Sincerely, on behalf of all the authors,

Jasper Landman

**Reviewer 1:**

**Comment:** The authors have worked hard to address all of my comments and suggestions on the original submission. The paper is much clearer now, although it is still heavy going.

**Response:** We are pleased to hear that our efforts to make the manuscript more accessible have paid off. We would like to once again repeat our previous acknowledgements for the outstanding feedback that the reviewer provided us with, which allowed us to make this manuscript far more accessible to the reader. We hope that by addressing all the feedback the paper is now less heavy going.

**Comment:** Now that I understand better what the authors are trying to establish, it is still not clear to me why they need 'grand canonical ensembles' to compute the 'fold-change' of transcription rate in the case when a TF binds to competing sites on the DNA or to a protein inhibitor. In the attached pdf, I analyze this mechanism using standard biochemical kinetic equations and the quasi-steady state approximation on binding of the TF to an inhibitor and to the gene's URS. Oscillations arise quite naturally for reasonable values of the kinetic parameters. (I replace the explicit time delay by an intermediate protein between the mRNA and the TF.) Other readers besides me might wonder why this standard approach is faulty, in the authors' opinion.

**Response:** We believe that with the use of standard biochemical kinetic equations and the quasi-steady state approximation one should indeed be able to obtain the same results as we do in our grand canonical approach – so not a faulty approach at all. The reviewer indeed shows in the attached pdf that oscillations arise from a system of standard differential equations. We have chosen the grand canonical ensemble formulation, as it makes the link with a mechanistic adsorption problem more obvious. Moreover, the formalism lends itself well to more complex systems of species with more binding equilibria, something which would be very tedious (but definitely possible) to do with traditional reaction kinetics, requiring more parameters and more equations than using an equilibrium transcription factor binding framework. However, the investment in terms of more parameters and more equations in principle pays off by being able to predict absolute transcription rates, which has not been the goal of this work.

We would argue that it is not the use of traditional biochemical reaction kinetics itself, but rather the use of the Hill equation to simulate the effect of transcription factor sequestration, which is mechanistically incorrect.

To address this point, we have added an additional motivating paragraph to the text.

**Changes to the manuscript:**

- We added the following paragraph to page 3, line 220:

*“The grand canonical ensemble formulation is particularly well suited to deal with systems where many species can bind to different sites. In principle, the models we propose in this work can also be derived from the more conventional biochemical formulation based on multiple chemical equilibria and reaction kinetics, such as presented in the work of e.g., \citet{Klumpp2008}, at the cost of requiring more parameters. We have chosen the grand canonical ensemble formulation, as it makes the link with a mechanistic equilibrium adsorption problem obvious.”*

**Comment:** I would only insist on one clarifying correction. On lines 88-97, the authors acknowledge earlier demonstrations that competition-driven titration can create sufficient nonlinearity to generate self-sustained oscillations in negative-feedback circuits (referencing Kim & Forger #33 and three papers by Buchler #31, 32, 34). Then they say that "implementation is in all of these cases [35-40] based on a mechanistically incorrect mechanism: namely allosteric cooperative binding for which the Hill equation was derived." I have not checked publications [35-40], which may indeed rely on Hill function nonlinearities, but papers [31-34] are not 'mechanistically incorrect' in my opinion. (See also, the pdf I have attached to this review.) Maybe the authors mean to say something like this: "Previous authors have shown that competition-driven titration effects can lead to highly nonlinear (ultrasensitive) response curves [31, 32, 34] and to oscillations when embedded in a negative-feedback loop [33]. However, in many cases [35-40] such 'titration oscillators' are based on mechanistically incorrect assumptions, namely, allosteric cooperativity leading to Hill function nonlinearities. Kim and Tyson [41] level similar criticisms at the use of standard quasi-steady state approximations to derive Michaelis-Menten kinetics in complex networks of interacting proteins."

If what I say is correct, then the authors need to address how their approach improves on the analysis of competition-driven ultrasensitivity analyzed in [31-34].

**Response:** Papers [31-34] model the effect of titration oscillators using traditional reaction kinetics. By committing to a fully kinetic model, these papers indeed find oscillations in a method that is mechanistically correct. Papers [35-40] are based instead on equilibrium models for transcription initiation, and use a Hill isotherm to generate the necessary nonlinearities. The advantages of using equilibrium models over fully kinetic models have been described elsewhere (e.g. Bintu et al., Curr. Op. Gen. Dev. 2005), and include the necessity for fewer equations and fitting parameters. Our work shows how titration oscillators can be analysed using an equilibrium transcription binding framework.

We agree with the reviewer that the text of this paragraph does not make that distinction clear, and we have revised it to emphasize the distinction.

**Changes to the manuscript:**

- The paragraph starting on line 88 now reads:

*“Although the notion exists that a competition-driven titration effect can also lead to sufficient nonlinearity to generate self-sustained oscillating reactions --- such circuits are coined `titration oscillators'[31-34] --- equilibrium modelling of transcription factor binding in these circumstances has so far resorted to a mechanistically incorrect mechanism: namely allosteric cooperative binding for which the Hill equation was derived.[35-40]. Kim & Tyson [41] level similar criticism at the use of quasi-Steady State Michaelis Menten kinetics which also reduces to a Hill equation.”*

**Reviewer 2:**

**Comment:** In this paper Landman et al. use methods from statistical physics to develop a model of gene transcription. The major novelty in the approach lies in the fact that nonlinear functions that describe transcription rates are derived using statistical mechanics rather than by assuming functional forms a priori (e.g. Hill functions).

The manuscript is not written in an accessible enough manner. In my opinion, too much prior knowledge of statistical mechanics is assumed by the authors (given the nature of the PloS Comp Biological readership). The criticisms of Reviewer 1 in the previous submission have not been adequately addressed.

**Response:** We are aware that the previous version of the manuscript was not accessible enough for the general readership of PLOS Computational Biology. With the revisions, we were very glad to hear that Reviewer 1 now considers the manuscript much clearer, although we agree that it could still be considered ‘heavy going’. We have made further changes throughout the manuscript to provide more direction and motivation in order to assist the reader.

However, we must respectfully disagree with the reviewer that the prior knowledge of statistical mechanics we assume is too much for the general readership of PLOS Computational Biology. Many well-cited works have appeared in PLOS Comp. Biol. or PLOS One (with even broader readership) in the past few years that feature similar levels of statistical mechanics (see e.g., Waldispühl, PLOS Comp. Biol. 2008, Möbius et al., PLOS Comp. Biol. 2010, Lecarpentier et al., PLOS Comp. Biol. 2015, Jones et al., PLOS One 2015, Bak et al., PLOS Comp. Biol. 2021).

In order to improve legibility, we have introduced a further motivating paragraph why we have chosen for the grand canonical ensemble. Furthermore, we have placed a sentence referring the reader to the step-by-step worked examples of a number of regulatory architectures that are available in the supplementary information.

**Changes to the manuscript:**

- We added the following paragraph to page 3, line 220:

*“The grand canonical ensemble formulation is particularly well suited to deal with systems where many species can bind to different sites. In principle, the models we propose in this work can also be derived from the more conventional biochemical formulation based on multiple chemical equilibria and reaction kinetics, such as presented in the work of e.g., \citet{Klumpp2008}, at the cost of requiring more parameters. We have chosen the grand canonical ensemble formulation, as it makes the link with a mechanistic equilibrium adsorption problem obvious.”*

- On page 4, line 342 we added the following paragraph:

*“In the supplementary information we have provided step-by-step worked examples on how to calculate the fold-change function $\Phi$ and the transcription factor fugacities $\lambda_i$ for a number of relevant regulatory architectures.”*

**Comment:** I found it difficult to formulate the proposed model in a manner that makes it comparable to the corresponding Hill function models. For example, in equation (18) Phi is a function of p whilst in equation 17 Phi is a function of many factors (e.g. free energies, fugacities). It has not been made clear how these are related. As many readers will not have a background in statistical mechanics, the interpretation and derivation of the fold change function (Phi) has not been made sufficiently clear.

**Response:** We thank the reviewer for bringing this unclarity to our attention. The fold-change is strictly a function that depends on the fugacities, binding free energies and additional free energy terms in the system. However, since the fugacities in turn are directly dependent on the protein concentrations through the mass balance equation (4), it is more convenient to write the fold-change directly as a function of the protein concentrations. This also helps the reader to see where the feedback loop in the system of DDEs comes into play.

We agree that we’ve been rather flexible with showing the exact dependency of the fold-change function (dependence on the binding free energies is for example always implicit). As such, we’ve decided to make this dependence more clear with an additional sentence when the fold-change function is introduced in the system of DDEs.

Furthermore, the supplementary information has a step-by-step description of how the fugacities depend on the protein concentrations in a number of relevant scenarios. As mentioned above, we added a paragraph pointing the reader to this section.

**Changes to the manuscript:**

- On page 6, line 421 we introduced the following sentences:

“The fold-change vector is implicitly a function of all the protein fugacities in the system, which in turn depend on the protein concentrations **p**, hence our choice to write $\Phi$ explicitly as a function of **p**.”

**Comment:** There were numerous statements made that did not include appropriate citation
(e.g. transcription factors such as LacI tend to be poorly soluble in water, the binding free energy of RNA polymerase to its specific site is relatively weak …)

**Response:** We apologise for this oversight, and have amended the text with the appropriate citations.

**Changes to the manuscript:**

- On line 285 we added the citation to DeHaseth et al., Biochemistry 1977
- On line 333 we added the citation to Garcia & Phillips, PNAS 2011

**Comment:** It is my view that this article, in its current formulation, does not fall within the remit of PloS Computational Biology. It is not clear what profound biological insights come from the work.

**Response:** with the changes outlined in the responses to all reviewers, we are confident that the manuscript now clearly shows what the biological insights of this work are, and that the manuscript is now suitable for publication in PLOS Computational Biology.

**Reviewer 3:**

**Comment:** Oscillatory genetic networks are at the heart of circadian rhythms. Understanding the conditions under which genetic networks can oscillate is therefore an important biological question. It is well known that cooperative binding of transcription factors to the DNA can yield the required non-linearity for oscillations. However, titration can also provide the required non-linearity. Yet, a thorough theoretical description and analysis have been lacking. In their manuscript, Landman and coworkers provide such a description and analysis. They use their description to derive under which conditions titration can induce oscillations. They show that the required non-linearity can arise via the binding of transcription factors to titration sites, by having multiple gene copies, and by having auxiliary sites forming DNA loops. Moreover, the show what the minimum number of binding sites and gene copies, and the maximum looping free energy, are for obtaining oscillations. These are important results that are of wide interest. The manuscript is also clearly written and the figures are beautiful and clear. I therefore believe that publication of this manuscript in PLCB is warranted.

While I like the efforts of the authors to apply their model to existing experimental data (the Stricker data) , I also believe that their work can be seen as predictions for new experiments. Figs. 5c-e provide nice predictions that could be tested experimentally. In particular, titration sites could be introduced into the genome.

**Response:** We are pleased with the very positive comments about our work.

**Comment:** My most important comment is:

- I am puzzled by the fluctuations in the delay. Are these dynamic fluctuations of the delay in time? If so, on what timescale do the delays fluctuate? Or are these fluctuations arising from cell-to-cell variability, which are static on the timescale of the oscillations? It is also not clear to me how these fluctuations are accounted for in the stability analysis - via simulations (direct integration of DDE), or via the theoretical stability analysis (but if so, how)?

**Response:** The fluctuations in the delay are indeed dynamic fluctuations of the delay in time, of roughly the same timescale as over which protein and mRNA levels vary. Our treatment of these fluctuations is admittedly fairly basic and is based purely on how much the stability contour shifts when a fluctuation of a certain magnitude is encountered. We aim to address the nature of fluctuations in titration oscillators in more detail in a future publication.

**Changes to the manuscript:**

- On page 10, line 655, we changed the first sentence of the paragraph about fluctuations to: “Fluctuations in $\gamma \tau$ *temporarily* shift the stability contour locally:”
- On page 10, line 674, we added the following sentence: “This shift was calculated by simply taking the position of the stability contour at $0.8 \gamma\tau$, and does not reflect a direct integration of stochastic differential equations.”

**Comment:**

- P_i is not defined before or immediately below Eq. 4 (but only much later)

- Eq. 4 is presented as a self-consistency equation, but that it is not directly from the equation itself (a self-consistency equation has the variable typically on both sides of the equality, but this is not immediately apparent here).

**Response:** We thank the referee for spotting this missing definition. Furthermore, the self-consistency comes from the fact that the fugacity is in principle a function of transcription factor copy number, although the exact functional form is not obvious. We find it by requiring the expected number of transcription factors over the different reservoirs to be consistent with the actual transcription factor copy number.

**Changes to the manuscript:**

- On page 3, line 272, we added the following sentence: *“where the fugacity needs to be chosen such that the expected number of transcription factors $i$ over the different reservoirs is self-consistent with $P_i$, the copy number of that protein in the cell.”*
- Removed the words “self-consistently” in the line immediately before eq. (4), page 3 line 269.

**Comment:** - Introduction, 2nd paragraph below Eq, 4, the interpretation of the fraction θ(λ1,λ2,...) / θ(λ1,0,...) only becomes clear after the reader is informed to which species 1, 2, etc. correspond to. It would be good to iterate that RNAP corresponds to species 1, and that the other indices refer to the transcription factors (TFs).

**Changes to the manuscript:**

- On page 3, line 295, we added the following sentence: *“We reiterate here that the index number 1 is reserved for RNAP, and the numbers 2 and up correspond to the transcription factors.”*

**Comment:** - I like the grand-canonical description, but in practice it is not very different from the more conventional description developed by Hwa and coworkers, where the partition function is written as a sum of exponents of [x] / ΚD, where [x] is the concentration of the transcription factor in the cytoplasm, which can be thought of as the fugacity, and KD is given by e^{-βε). In both cases, one needs to solve a constraint, which indeed arises from a mass balance. Perhaps the authors could briefly comment on this viewpoint.

**Response:** Indeed, the method developed by Hwa et al. should give essentially the same results. We prefer the grand canonical formalism as it makes the link to a mechanistic equilibrium problem more obvious in our view.

**Changes to the manuscript:**

- We added the following paragraph to page 3, line 220:

*“The grand canonical ensemble formulation is particularly well suited to deal with systems where many species can bind to different sites. In principle, the models we propose in this work can also be derived from the more conventional biochemical formulation based on multiple chemical equilibria and reaction kinetics, such as presented in the work of e.g., \citet{Klumpp2008}, at the cost of requiring more parameters. We have chosen the grand canonical ensemble formulation, as it makes the link with a mechanistic equilibrium adsorption problem more obvious.”*

**Comment:** - While I think for this work it is fine to assume that the mRNA and protein degradation rates are equal, in many systems the mRNA lifetime is shorter than the cell-division time, which means that degradation is the dominant mechanism for decay, not dilution by growth.

**Response:** In this manuscript we’ve taken both degradation rates to be of comparable magnitude, but this assumption goes without loss of generality. While it would be interesting to look at the case of more extreme difference between mRNA and protein degradation rates, we believe it falls outside the scope of the current manuscript.

**Changes to the manuscript:**

- on page 5, line 373, we changed the sentence: *“However, often the dominant mechanism is (…)” to “However, in many cases the dominant mechanism is (…)”*

**Comment:** - I find it intuitive that titration sites can induce the required non-linearity for generating oscillations. The result that just having more than one gene copy is sufficient for inducing oscillations is less intuitive to me (Fig. 2). What is the minimal gene copy number at which oscillations arise? And is the behavior of this system then highly similar if not identical to that of a single gene but with an identical number of TF binding sites? Or does the fact that each TF binding sites comes with a promoter and its adjacent gene (which leads to the production of proteins) make a big difference? I presume panels c and d of Fig. 5 give the answer – to get the required sharpness of |dΦ/dp| ~ 4 οr higher, 20 competitor sites would be required for a single gene, while a 100 copies of identical genes would be required. So there is indeed a difference. Is this interpretation correct?

**Response:** The referee points out an interesting effect here. Yes, there is a difference between the situation of N identical copies of the same gene, and a single gene with N-1 competing binding sites of the same binding free energy, but this difference originates from the fact that those N identical copies all produce mRNA. As a consequence, while the titration curve is mathematically equivalent, the larger mRNA production rate shifts the stationary point of this system further along the curve.

A further difference is that the binding free energy of competing sites can (and most probably does) differ from the binding free energy of the operator sites of the gene of interest. In general, stronger binding competitor sites provide a steeper titration effect.

**Changes to the manuscript:**

- On page 10, line 698, we added the following paragraph to the discussion:

*“While it is perhaps less intuitive that multiple copies of the same gene can lead to a titration effect similar to the situation with competitor sites, mechanistically the titration effect in both cases is the same: the existence of one or more specific sites depletes the reservoir of free transcription factors. A reservoir of $N$ gene copies lead to the same titration curve as $N-1$ sites competing with a single gene copy --- provided the binding free energy of the competing sites is the same. However, there is one subtle difference: the additional production terms arising from the multiple gene copies shift the stationary point of the system to a different point along the titration curve. As such, one can find the optimal conditions for self-sustained oscillations at a different $P^{(0)}$ per gene.”*

**Comment:** - line 569: "such the real" -> "such that the real"

**Changes to the manuscript:**

- this suggestion was taken over.

**Comment:** - Caption Fig. 5a: For ease of reading, please explain in the caption what is on the axes.

**Changes to the manuscript:**

- Added the following sentence to the caption of Fig. 5: “A map of stable oscillations in the phase space of normalised delay time $\gamma \tau$ versus the slope of the fold-change in the stationary point of the system $\mathrm{d}\Phi/\mathrm{d}p (m^*,p^*)$.”

**Comment:** - line 611: "We show the positive branch of the real part of λ in Fig. 5a". I think I see what the authors mean, but this sentence is confusing, I feel. The contourplot is a contourplot of Im{λ}. The white dashed line is the border between the oscillatory and the non-oscillatory regime, which is the line where the real part of the dominant eigenvalues λ equals 0 - this is clear. But what do the authors mean by "positive branch of the real part" in this sentence (line 611)?

**Response:** The wording “positive” here is indeed incorrect, given that outside of the stability contour the dominant roots have negative real parts.

**Changes to the manuscript:**

- We changed the line in the caption of Fig. 5a to: “The contours indicate the magnitude of the imaginary part of the dominant root $\lambda$, (…)”
