# Supplementary material for: Transcription factor competition facilitates self-sustained oscillations in single gene genetic circuits
Source: PLoS Comput Biol. 2023 Sep 29;19(9):e1011525. doi: 10.1371/journal.pcbi.1011525 (PMC10566692; doi:10.1371/journal.pcbi.1011525)
Supplement: Attachment — Submitted filename: ResponseReviewers_20230913.docx [file pcbi.1011525.s007.docx]

Utrecht, September 13, 2023

Dear professor Mendes,

We were pleased to receive the very constructive referee feedback report on our manuscript PCOMPBIOL-D-23-00261. Below we’ve listed the referee responses, along with our response and corresponding changes in the manuscript. With these changes we hope that our manuscript is now acceptable for publication in PLOS Computational Biology.

Sincerely, on behalf of all the authors,

Jasper Landman

**Reviewer 1**

**Comment:** The authors have adequately addressed all of the comments and suggestions of the Reviewers, so I am willing to recommend publication. However, I have a few comments for the authors and the editor, which may require some minor changes.

**Response:** We are glad to hear that the reviewer considers their comments and suggestions adequately addressed, and are happy to take into account the final comments provided.

**Comment:** The authors make clear in their response to me that their complaint is with using a Hill function to describe the effect of TF sequestration, not with using standard biochemical reaction kinetic equations. I agree with them that Hill functions are not appropriate in this context, but I would argue that the standard kinetic approach is much simpler than the grand canonical approach introduced by the authors. There is no difficulty with applying the standard approach to more complex systems with multiple species involved in many binding reactions: standard numerical methods of solving differential algebraic equations (DAEs) handle such problems easily and accurately. I don’t understand how linking the grand canonical approach to ‘mechanistic adsorption problems’ is an advantage for computational systems biologists. I don’t think the authors provide sufficient motivation to readers to learn the methodology of grand canonical ensembles. If standard kinetic rate equations are OK, why make life difficult?

This is one case in which I think it may be advantageous to publish an addendum to the paper, making public some of the interchanges between the authors and reviewers (suitably edited).

**Response:** We respectfully disagree that the standard biochemical reaction kinetic approach is much simpler than the grand canonical approach. There are advantages and disadvantages to both approachess, as we briefly discuss in our manuscript, and the chosen description is mainly a matter of taste.

We absolutely agree with the referee that it would be advantageous to publish the peer review process of this manuscript along with the final article – in fact, we had already decided that we would like to make use of the option of PLOS Comp. Biol. to publish the peer review process along with the article. This is one example where the very extensive peer review reports have considerably improved the quality of the manuscript.

**Comment:** Regarding the change proposed to the paragraph starting on line 88:

*Although the notion exists that a competition-driven titration effect can also lead to sufficient nonlinearity to generate self-sustained oscillating reactions --- such circuits are coined `titration oscillators'[31-34] --- equilibrium modelling of transcription factor binding in these circumstances has so far resorted to a mechanistically incorrect mechanism: namely allosteric cooperative binding for which the Hill equation was derived.[35-40]. Kim & Tyson [41] level similar criticism at the use of quasi-Steady State Michaelis Menten kinetics which also reduces to a Hill equation.”*

I don’t agree with the blue change. Although I haven’t checked all references 35-40, I don’t think the authors actually derived a Hill function from ‘equilibrium modeling’ of TF binding, but rather they assumed that a Hill function would adequately describe the situation. This assumption may be mechanistically incorrect, but other more recent papers [31-34, 41] apparently use singular perturbation methods to get mechanistically correct and accurate approximations for ‘titration effects’. I think the authors should be more accurate in what they say about these papers [31-40], especially so when leveling criticisms.

**Response:** We apologize for the unclarity in the revised text. The word ‘derived’ referred to the original work of Hill [27], and not to the work presented in refs [35-40], but we recognize that this ambiguity is present. In fact, Hill himself also didn’t actually derive the equation, but assumed its functional form, which we already mention in line 85-88. Consequently, we realized that we could considerably simplify the blue text.

**Changes to the manuscript:**

On lines 107-111 we changed the paragraph to:

*“Although the notion exists that a competition-driven titration effect can also lead to sufficient nonlinearity to generate self-sustained oscillating reactions --- such circuits are coined `titration oscillators'[31-34] --- equilibrium modelling of transcription factor binding in these circumstances has so far resorted to the use of mechanistically incorrect Hill equation. [35-40] Kim & Tyson [41] level similar criticism at the use of quasi-Steady State Michaelis Menten kinetics which also reduces to a Hill equation. Nonequilibrium modelling of titration oscillators can lead to mechanistically correct results, at the cost of requiring more parameters.”*
